# Early prediction of microvascular obstruction prior to percutaneous coronary intervention

**DOI:** 10.1038/s41598-025-94528-7

**Published:** 2025-03-19

**Authors:** Ziyu Zhou, Qing Chen, Zeqing Zhang, Tingting Wang, Yan Zhao, Wensu Chen, Zhuoqi Zhang, Shuyan Li, Boming Song

**Affiliations:** 1 Information Center, Chengdu Second People’s Hospital, Chengdu, 610017 China; 2 Department of Cardiology, Huai’an First People’s Hospital, Nanjing Medical University, Huai’an, 223300 China; 3 Department of Cardiology, The Affiliated Hospital of Xuzhou Medical University, Xuzhou, 221002 Jiangsu China; 4 The First School of Clinical Medicine, Xuzhou Medical University, Xuzhou, 221002 Jiangsu China; 5 School of Medical Information and Engineering, Xuzhou Medical University, Xuzhou, 221002 Jiangsu China

**Keywords:** Percutaneous coronary intervention, Acute myocardial infarction, Microvascular obstruction, Machine learning, Logistic regression, Cardiology, Bioinformatics

## Abstract

Early prediction of microvascular obstruction (MVO) occurrence in acute myocardial infarction (AMI) patients undergoing percutaneous coronary intervention (PCI) can facilitate personalized management and improve prognosis. This study developed a prediction model for MVO occurrence using preoperative clinical data and validated its performance in a prospective cohort. A total of 504 AMI patients were included, with 406 in the exploratory cohort and 98 in the prospective cohort. Feature selection was performed using random forest recursive feature elimination (RF-RFE), identifying five key predictors: High-Sensitivity Troponin T, Neutrophil Count, Creatine Kinase-MB, Fibrinogen, and Left Ventricular Ejection Fraction. Among the models developed, logistic regression demonstrated the highest predictive performance, achieving an AUC score of 0.800 in the exploratory cohort and 0.792 in the prospective cohort. This model has been integrated into a user-friendly online platform, providing a practical tool for guiding personalized perioperative management and improving patient prognosis.

## Introduction

Ischemic heart disease (IHD) is a major cause of death worldwide, accounting for 12.7% of all deaths^1^. Acute myocardial infarction (AMI), as a severe type of IHD, poses a serious threat to human health^2,3^. Timely percutaneous coronary intervention (PCI) to open the infarct-related artery can salvage ischemic myocardium, reduce infarct size, and decrease complications and mortality after infarction^4^. However, reperfusion injury is unavoidable. Microvascular obstruction (MVO) is a common phenomenon that occurs after mechanical reperfusion in patients with STEMI^5,6^, associated with dysfunction of the microvascular system and failure of reperfusion in the microcirculation^7^. The incidence of MVO in patients with AMI was 40-50% according to previous studies^8,9^. MVO is strongly associated with adverse left ventricular (LV) remodeling^10^ and serves as an independent predictor of major adverse cardiovascular events (MACE)^11–13^, significantly influences patient prognosis^14^. Persistent MVO can lead to intramyocardial hemorrhage (IMH), exacerbating adverse patient outcomes^15^. Therefore, early prediction of MVO risk and timely intervention are particularly important.

Cardiac magnetic resonance (CMR) is currently the most commonly used non-invasive method to accurately identify MVO^6,16–19^. However, due to the confined space and high costs associated with CMR, it is paramount to establish an automated prediction model for MVO based on patients’ preoperative information. This model helps clinicians early identify high-risk populations for postoperative MVO and provides guidance for implementing targeted measures in medication and postoperative management, ultimately improving patient prognosis.

With the development of big data analysis and machine learning technologies, building models to predict disease risks using patients’ readily available diagnostic information has become feasible. In the field of cardiovascular disease assessment, studies have applied machine learning models to predict in-hospital bleeding^20^, contrast-induced acute kidney injury^21,22^, and evaluate the predictive value for MACE after PCI in patients with AMI^23,24^, improving risk stratification in these patients^25^. These models integrate patients’ demographic information, laboratory and imaging findings, and so on, enabling noninvasive individualized prediction of patient prognosis after AMI.

This study aims to develop a multi-factor prediction model for the risk of MVO after PCI. The model is entirely reliant on the patient’s baseline information and various clinical examination indicators before PCI. Its objective is to achieve automated assessment and early identification of high-risk MVO patients, optimizing clinical treatment and medication plans.

## Methods

### Study design

This study initially retrospectively included data from patients diagnosed with AMI in the Department of Cardiology at the Affiliated Hospital of Xuzhou Medical University between January 2018 and December 2022. These patients underwent CMR within one week after primary PCI. Their data were used to train the MVO prediction model and select the best-performing model. Subsequently, a prospective cohort from January to August 2023 was gathered to validate the model. The best model was then employed to develop an online prediction platform.

### Ethics approval

In accordance with the Helsinki Declaration, the Ethics Committee of Xuzhou Medical University Affiliated Hospital has granted approval for this study (Ethics Number: XYFY2023-KL487). Patient informed consent has been waived for this research as per the committee’s authorization.

### Study participants

**Fig. 1:**
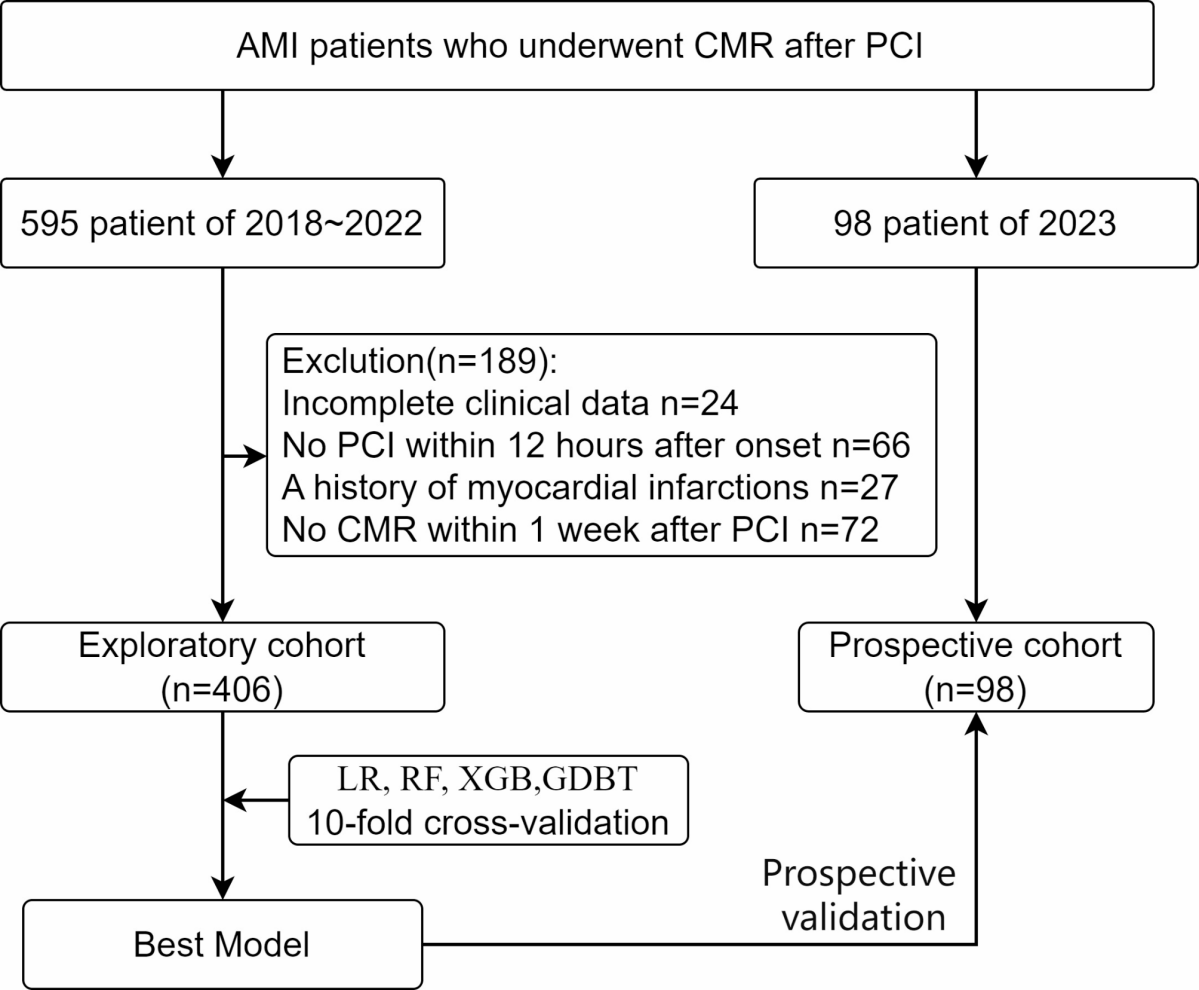
Flow diagram of patient selection.

This study included 595 patients diagnosed with AMI who underwent CMR within 1 week after PCI From July 2019 to December 2022. All patients exhibited normal blood flow restoration in the postoperative period. We excluded patients with incomplete clinical data, who did not undergo PCI within 12 h after onset, who had old myocardial infarction, and who did not undergo CMR within 1 week after surgery. To validate the model’s effectiveness, data were prospectively collected from patients in 2023. As illustrated in Fig. 1, a total of 406 patients were included in the exploratory cohort, along with an additional 98 patients in the prospective cohort.

### Predictor variables

This study gathered preoperative clinical data from patients through the hospital’s electronic medical record system, encompassing clinical information, blood tests, and echocardiographic data. In total, 29 clinical features were ultimately incorporated into the analysis.

### Clinical data

Patient clinical data were collected, including age, gender, weight, height, body mass index (BMI), Killip classification, and history of hypertension and diabetes. Specifically, for gender, males were recorded as 1 and females as 0. For Killip classification, grade I/II was coded as 0 and grade III/IV was coded as 1.

### Laboratory data

Various laboratory data were gathered from patients, encompassing glycemic indices (glycated hemoglobin (HbA1c), fasting blood glucose (FBG)), lipid indices (total cholesterol (TC), triglyceride (TG), low-density lipoprotein (LDL-C), lipoprotein(a) (LP(a))), inflammatory markers (white blood cell (WBC) count, neutrophil count, lymphocyte count, neutrophil-to-lymphocyte ratio (NLR), systemic immune-inflammatory index (SII), high-sensitivity C-reactive protein (hs-CRP), fibrinogen (FIB), D-dimer), myocardial injury markers (creatine kinase-MB (CK-MB), high-sensitivity cardiac troponin T (hs-TnT)), and cardiac function indicators (NT-proBNP). Among them, hs-CRP, CK-MB, hs-TnT, and NT-proBNP take the highest values during the hospital stay. All blood samples were collected within 24 h after admission and all indicators were tested in the hospital laboratory.

### Echocardiographic data

In this study, echocardiographic data was collected from patients, including left ventricular ejection fraction (LVEF) and left ventricular end-diastolic dimension (LVEDD). All patients underwent an echocardiographic examination within 48 h of admission.

### Outcome measures

The primary outcome measure was the occurrence of MVO. This study utilized CMR, which is considered the optimal diagnostic imaging modality for the detection of MVO, to identify the presence of MVO^17,18^. All enrolled patients underwent CMR examination within one week after PCI. CMR sequences mainly include contrast-enhanced steady-state free precession cine (CE-SSFP) sequences and late gadolinium enhancement (LGE) sequences. MVO appeared as a non-signal or hypointense region (depicted in black) within the infarct core (depicted in white) with high signal intensity on LGE images on CMR. Two independent cardiovascular internists interpreted CMR images to assess the presence of MVO. In cases of disagreement, a third senior physician made the final determination.

### Statistical analysis

Categorical data were expressed as numbers and percentages (%) and analyzed utilizing the chi-square test. The normal distribution of continuous data was assessed by the Kolmogorov-Smirnov test. Normally distributed continuous data were expressed as mean ± standard deviation and analyzed using the t-test. Non-normally distributed continuous data were presented as median (25th quartile, 75th quartile) and analyzed using the Mann-Whitney U test. Missing data were imputed by the random forest method using the missForest package in Python^26^. All data were analyzed for statistical significance utilizing the ‘SciPy’ library in Python 3.8, with a two-tailed p-value threshold set at less than 0.05.

### Feature selection

Feature selection is a critical step in the modeling process, aiming to identify the variables with the maximum information for predictive modeling and enhance generalization performance. In this study, Random Forest Recursive Feature Elimination (RF-RFE) was employed to systematically assess and select the most important features from the dataset. RF-RFE is a recursive technique that utilizes the power of random forests to evaluate the importance of each feature and iteratively eliminate less significant variables^27^.

### Model building and validation

A systematic approach was employed for dataset partitioning, model construction, and validation. Training and validation utilized data from the exploratory cohort. Initially, the dataset was randomly shuffled to eliminate the bias related to the order of samples. A 10-fold cross-validation strategy was implemented, dividing the dataset into 10 equal folds. In each iteration of the cross-validation, nine folds were utilized for training the model while the remaining fold served as the test set. To ensure a fair evaluation of model performance, this process was repeated with 10 random shuffles, generating diverse training and testing splits.

For model construction, logistic regression (LR) and various machine learning algorithms, including Random Forest (RF), Extreme Gradient Boosting (XGB), and Gradient Boosting Decision Trees (GBDT), were employed. Bayesian optimization was used to identify the optimal hyperparameters for each algorithm, thereby enhancing model performance. Model performance was assessed based on the mean area under the receiver operating characteristic curve (AUROC) from 10 × 10 iterations, providing a threshold-independent measure of model discrimination.

To validate the robustness of the model, data from the prospective cohort were collected. The models trained on the exploratory cohort were applied to the prospective cohort, where performance was evaluated using metrics including accuracy, sensitivity, specificity, and AUC. Additionally, Decision Curve Analysis (DCA) was referenced to assess the clinical utility of the models. This comprehensive evaluation ensured a thorough assessment of the model’s effectiveness.

### Online prediction platform

In order to integrate the MVO risk prediction model into clinical practice, the best-performing model was developed into an online prediction platform. This platform allows doctors to quickly enter the preoperative medical information of patients and predict the risk of MVO. To ensure enhanced user experience, the website was optimized for mobile devices. The application, developed using HTML, CSS, and Python, includes the necessary Python dependencies as listed in Table 1.

**Table 1:** The necessary Python dependencies.

Dependencies	Versions
Python	3.8.12
Flask	3.0.2
Scikit-learn	1.3.2
Gunicorn	21.2.0
Numpy	1.24.4

## Results

### Patient characteristics

This study involved a total of 504 participants, comprising 406 individuals in the training set cohort and 98 in the prospective validation cohort. Within the training set cohort, 213 individuals (52.5%) were identified with MVO, and Their baseline characteristics are presented in Table 2. In the prospective cohort, 55 individuals (56.1%) were identified with MVO, and Their baseline characteristics are summarized in Table 3.

**Table 2:** Clinical features between different groups in the exploratory cohort.

Variables	MVO (*n* = 213)	No MVO (*n* = 193)	*P* value
Clinical variables			
Age, (years)	55.6 ± 12.2	57.6 ± 12.0	0.104
Male, n (%)	187(88)	153(79)	0.020
Weight, (kg)	72.0(65.0,82.0)	71.0(65.0,80.0)	0.484
Height, (cm)	170.0(165.0,173.0)	170.0(165.0,173.0)	0.715
BMI, (kg/m2)	25.8 ± 3.7	25.6 ± 3.4	0.493
Hypertension, n (%)	92(43)	85(44)	0.496
Diabetes mellitus, n (%)	56(26)	48(25)	0.595
Killip grade			0.126
grade I/II, n (%)	205(96)	176(91)	
grade III/IV, n (%)	8(4)	17(9)	
Laboratory variables			
WBC count, (10*9/L)	10.0(8.3,11.9)	8.6(7.3,10.2)	< 0.001
Neutrophil count, (10*9/L)	7.8(6.3,9.9)	6.4(5.3,8.0)	< 0.001
Lymphocyte count, (10*9/L)	1.4(1.0,1.7)	1.4(1.1,1.9)	0.462
Platelet count, (10*9/L)	214.3 ± 62.2	210.6 ± 54.1	0.521
TyG index	3.8(2.7,6.8)	4.6(3.0,6.7)	0.360
NLR, (%)	5.6(4.2,7.9)	4.5(3.2,6.5)	< 0.001
SII, (10^9)	1192.3(842.8,1833.4)	908.9(620.5,1443.6)	< 0.001
Fib, (g/L)	2.8(2.3,3.7)	2.5(2.2,3.2)	0.002
D-dimer, (ug/mL)	0.2(0.0,0.4)	0.2(0.0,0.3)	0.407
Hs-CRP	28.0(13.5,62.7)	17.7(8.5,38.8)	< 0.001
HbA1c, (%)	5.8(5.5,6.9)	5.8(5.5,6.4)	0.242
FBG, (mmol/L)	5.8(5.0,7.8)	5.6(4.9,6.7)	0.023
TC, (mmol/L)	4.4 ± 1.0	4.5 ± 1.0	0.192
TG, (mmol/L)	1.3(0.9,2.0)	1.5(1.1,2.1)	0.017
LDL-C, (mmol/L)	2.8 ± 0.8	2.9 ± 1.0	0.069
LP(a), (mg/L)	201.0(139.0,312.0)	220.0(140.0,339.0)	0.332
CK-MB, (ng/mL)	195.5(107.0,300.0)	73.5(25.7,198.0)	< 0.001
NT-proBNP, (pg/mL)	1682.4(916.5,2862.0)	1225.0(619.0,2002.0)	< 0.001
Hs-TnT, (ng/L)	4111.0(2137.0,7475.0)	1431.0(589.0,3334.0)	< 0.001
Echocardiographic variables			
LVEF, (%)	51.0(49.0,54.0)	56.0(51.0,59.0)	< 0.001
LVEDD, (mm)	51.0(48.0,53.0)	49.0(47.0,52.0)	< 0.001

BMI, body mass index; WBC, white blood cell; TyG index, the triglyceride-glucose index; NLR, neutrophil-to-lymphocyte ratio; SII, systemic immune-inflammatory index; Fib, fibrinogen; HbA1c, glycated hemoglobin; FBG, fasting blood glucose; TC, total cholesterol; TG, triglyceride; LDL-C, low-density lipoprotein; LP(a), lipoprotein(a); NT-proBNP, N-terminal pro-B-type natriuretic peptide; CK-MB, creatine kinase-MB; hs-TnT, high sensitivity troponin T; LVEF, left ventricular ejection fraction; LVEDD, left ventricular end-diastolic dimension. Data are presented as the mean ± SD, median (IQR), or n (%).

**Table 3:** Clinical features between different groups in the prospective cohort dataset.

Variables	MVO (*n* = 55)	No MVO (*n* = 43)	*P* value
Clinical variables			
Age, (years)	55.7 ± 13.3	58.3 ± 11.6	0.329
Male, n (%)	48(87)	35(81)	0.020
Weight, (kg)	75.0(70.0,85.5)	73.0(65.5,80.0)	0.075
Height, (cm)	170.0(165.0,175.0)	168.0(163.5,173.0)	0.323
BMI, (kg/m^2^)	27.1 ± 3.4	26.0 ± 3.3	0.117
Hypertension, n (%)	26(47)	27(63)	0.496
Diabetes mellitus, n (%)	13(24)	7(16)	0.595
Killip grade			0.126
grade I/II, n (%)	53(96)	43(100)	
grade III/IV, n (%)	2(4)	0(0)	
Laboratory variables			
HbA1c, (%)	5.7(5.5,6.1)	5.8(5.5,6.2)	0.409
FBG, (mmol/L)	5.8(5.4,6.7)	5.6(5.1,6.0)	0.275
TC, (mmol/L)	4.5 ± 0.8	4.5 ± 1.1	0.739
TG, (mmol/L)	1.5(1.0,2.3)	1.7(1.1,2.0)	0.83
LDL-C, (mmol/L)	2.8 ± 0.6	2.8 ± 1.0	0.848
LP(a), (mg/L)	186.0(99.0,276.5)	210.0(132.0,360.5)	0.133
TyG index	4.9(3.1,7.2)	4.8(3.1,6.0)	0.694
WBC count, (10*9/L)	10.2(7.8,11.9)	9.2(8.1,11.2)	0.235
Neutrophil count, (10*9/L)	7.5(5.9,9.5)	7.0(5.6,8.2)	0.142
Lymphocyte count, (10*9/L)	1.5(1.1,1.9)	1.6(1.3,2.2)	0.202
Platelet count, (10*9/L)	214.9 ± 60.0	221.5 ± 50.1	0.563
NLR, (%)	4.9(3.7,7.0)	4.1(2.9,5.2)	0.033
SII, (10^9)	1032.5(776.9,1441.4)	834.4(693.0,1159.6)	0.066
Fib, (g/L)	2.9(2.4,3.3)	2.8(2.4,3.4)	0.883
D-dimer, (ug/mL)	0.1(0.0,0.4)	0.1(0.0,0.3)	1.000
Hs-CRP, (mg/L)	37.1(18.6,79.0)	22.7(8.2,60.8)	0.022
CK-MB, (ng/mL)	270.0(106.0,300.0)	79.8(19.6,166.0)	< 0.001
NT-proBNP, (mg/L)	1429.0(893.5,2472.4)	993.0(576.5,1549.0)	0.037
Hs-TnT, (ng/L)	4094.0(2825.5,8063.5)	1632.0(652.0,2971.0)	< 0.001
Echocardiographic variables			
LVEF, (%)	52.0(48.0,56.0)	57.0(52.0,60.0)	< 0.001
LVEDD, (mm)	49.0(47.0,52.0)	49.0(47.0,51.0)	0.371

### Feature selection

**Fig. 2:**
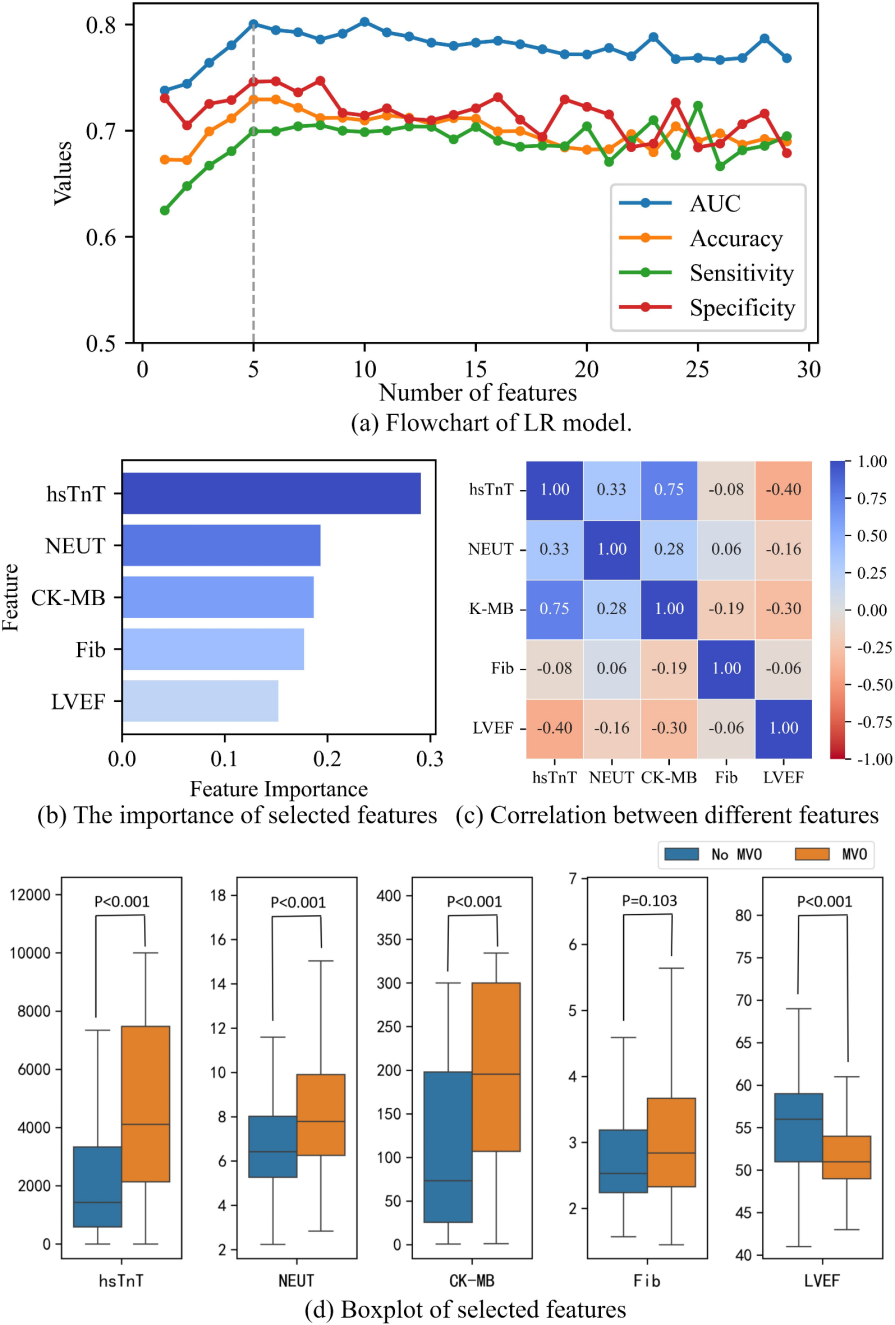
Feature selection and analysis.

Using RF-RFE, an iterative process was employed to eliminate the least important features. Figure 2a illustrates the performance of the LR model with varying numbers of retained features.

In order to achieve better model performance with a minimal set of variables, a total of 5 preoperative variables were identified as predictors of MVO. These features include hs-TnT, NEUT, CK-MB, Fib, and LVEF. The importance of the selected features is illustrated in Fig. 2b. The most crucial feature is hs-TnT. The Pearson correlation coefficient between selected features is depicted in Fig. 2c. The figure demonstrates a low correlation among the selected features, indicating the absence of unnecessary redundant features. Figure 2d displays boxplots of the selected features. The data in the figure exhibits a skewed distribution, and among the MVO and No MVO groups, there are significant differences in the other four features, excluding Fibrinogen.

### Model performance

For the exploratory cohort, the accuracy, sensitivity, specificity, and AUC of the LR model in the interactive test dataset are 0.729, 0.751, 0.709, and 0.800, respectively.

**Fig. 3:**
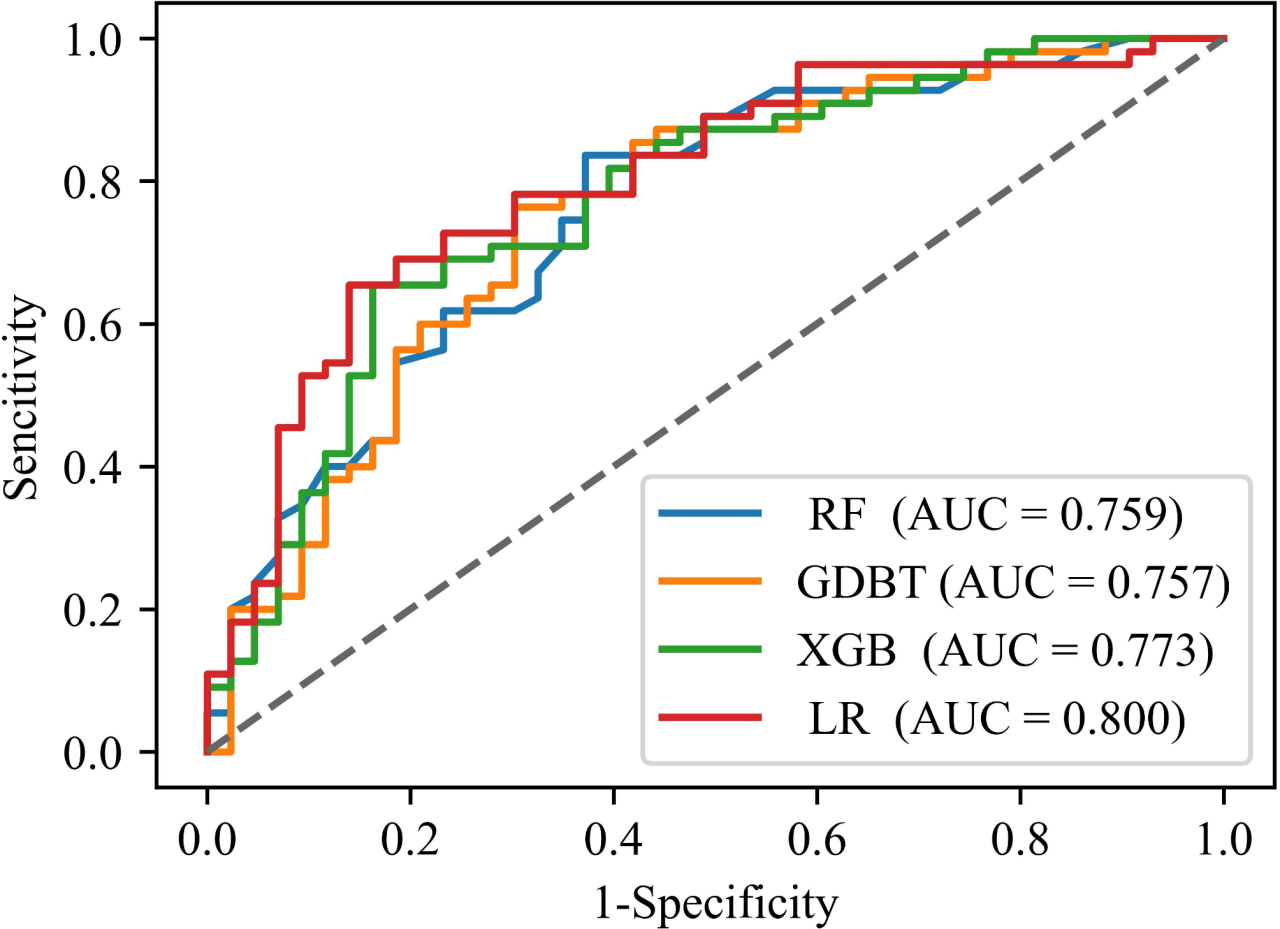
ROC curves for different models on the test dataset. RF: Random Forest; GDBT: Gradient Boosting Decision Trees; XGB: Extreme Gradient Boosting Decision Trees; LR: logistic regression.

Additionally, the performance comparison between three machine learning algorithms and LR algorithms was conducted in the test dataset. The machine learning algorithm models were constructed in the exploratory cohort and utilized the Bayesian optimization algorithm to search for the optimal hyperparameters. As depicted in Fig. 3, the LR model achieved the best AUC score of 0.800.

To validate the effectiveness of the models, the machine learning model and the LR model were compared again in the prospective cohort, as shown in Table 4. The results indicated that the LR model consistently outperformed the other models.

**Table 4:** Performance of different models on the prospective cohort dataset.

	Accuracy	Sensitivity	Specificity	AUC
RF	0.714	0.628	0.782	0.758
GDBT	0.724	0.698	0.745	0.763
XGB	0.673	0.628	0.709	0.776
LR	0.714	0.698	0.727	0.792

**Fig. 4:**
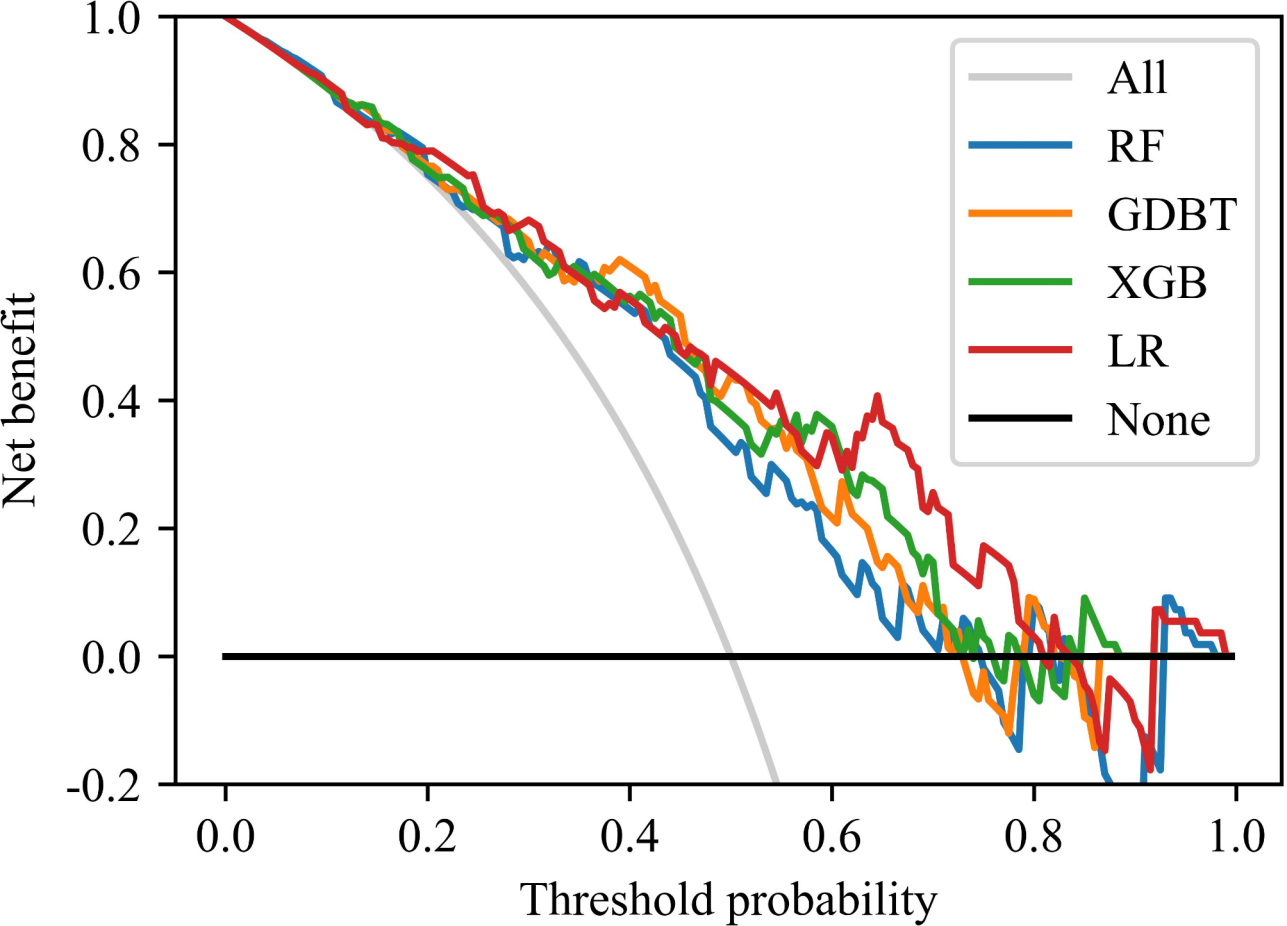
Decision curve analysis of the different models. In the figure, the colorful curve represents the predicted performance of the different models respectively. In addition, there are two lines, which represent two extreme cases. The gray vertical line represents the hypothesis that all patients have MVO; the black horizontal line represents the hypothesis that non-MVO occurs.

The Decision Curve Analysis (DCA) corresponding to the prospective cohort dataset is depicted in Fig. 4. The curve indicates that this LR predictive model could effectively distinguish MVO for clinical decisions when the MVO probability was between 0.2 and 0.8. Within reasonable threshold probabilities, the LR model achieves a higher benefit.

### Online platform

**Fig. 5:**
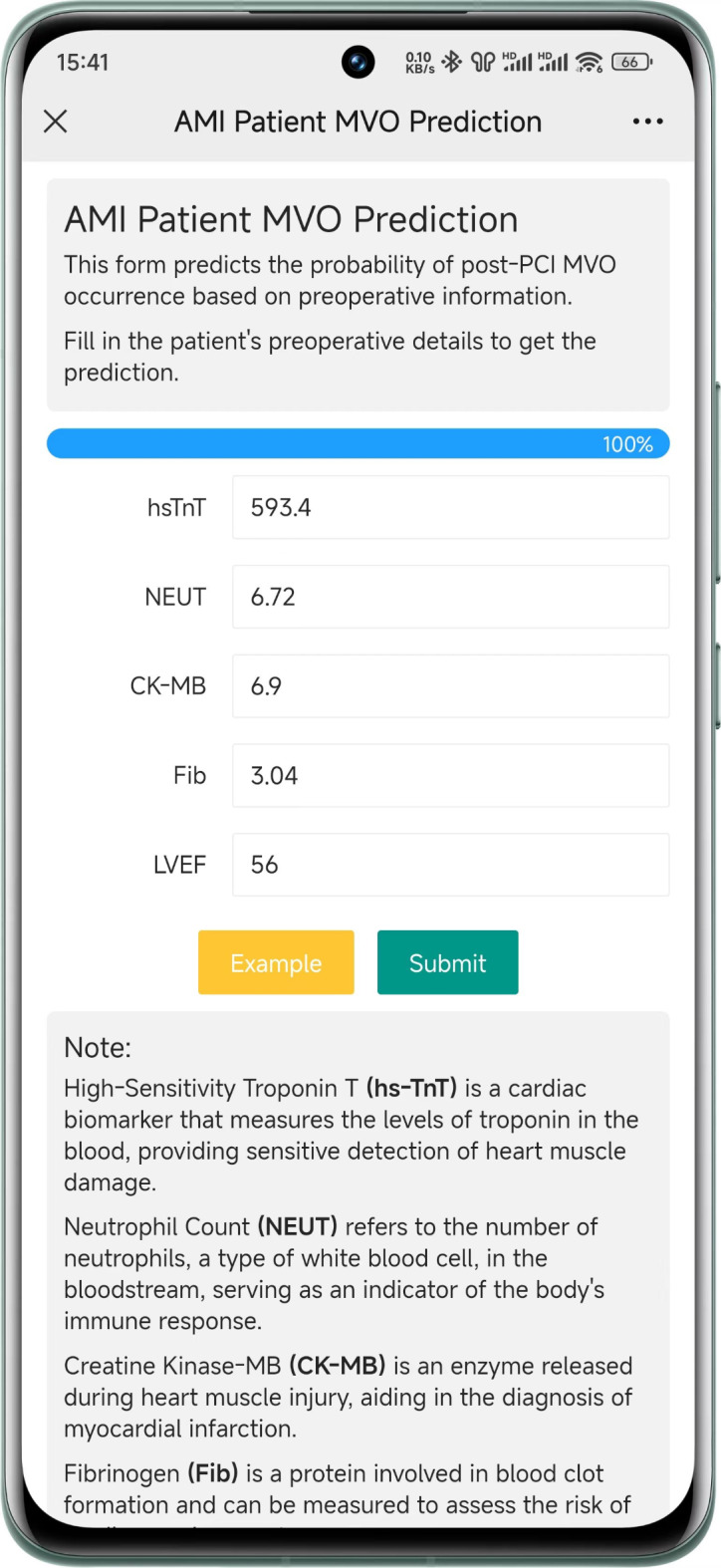
The online platform of the MVO prediction model.

We have developed a mobile-friendly online platform using the best model, enabling doctors to swiftly predict the risk of MVO occurrence in patients. The website provides a mobile-optimized interface (as shown in Fig. 5) allowing doctors to input five features (hs-TnT, NEUT, CK-MB, Fib, and LVEF) and promptly receive a prediction of the probability of MVO occurrence. It serves a vital role in identifying high-risk MVO populations before PCI, assisting in preoperative medication and perioperative management. This website can be accessed at https://mvo.model.smilemed.cn.

## Discussion

This study evaluated the predictive efficacy of four models (GBDT, RF, XGB, and LR) for MVO occurrence post-PCI. The findings revealed that the LR model outperformed the others. The LR model, based on five features (hs-TnT, NEUT, CK-MB, Fib, and LVEF), demonstrated good predictive performance in both cross-validation and prospective cohort datasets. These results suggest its potential utility in forecasting MVO after PCI in patients with AMI.

The RF-RFE algorithm was used for feature selection in our predictive model. The RF-RFE method effectively identified features suitable for constructing the LR model, providing a novel approach for LR to automatically recognize variable combinations most relevant to the outcomes.

The top five indicators associated with MVO were hs-TnT, NEUT, CK-MB, Fib, and LVEF. Hs-TnT and CK-MB are both crucial biomarkers for myocardial injury. However, hs-TnT demonstrates greater sensitivity in detecting mild myocardial damage compared to CK-MB. A study by Mathieu Schaaf et al. showed, compared with high-sensitivity troponin I (hs-TnI) and standard troponin I (s-TnI), peak hs-TnT had a better predictive ability for MVO^28^. The levels of hs-TnT measured at different time points have a certain correlation with MVO and similar predictive ability^29^. Previous studies have indicated that the levels of CK-MB are higher in the MVO group compared to the no MVO group ^22,30^. Consistent with these findings, our study reveals that the predictive efficacy for MVO is improved when combining hs-TnT and CK-MB.

Neutrophils are the cells that directly participate in the processes of inflammation and thrombosis, and their role at the site of local vascular injury is of crucial importance. In the early stage of myocardial ischemia/reperfusion (I/R) injury, neutrophils are activated under the action of inflammatory factors and reach the injured heart^31^. Activated neutrophils not only adhere to the vascular endothelium—a crucial process in microvascular injury^32,33^, but also obstruct microcirculatory blood vessels^34^. Additionally, their release of significant quantities of pro-inflammatory substances, including ROS, proteases, and lysosomal enzymes, can induce damage to the microvascular structure, resulting in edema in vascular endothelial cells and the formation of MVO^35^. Notably, the inhibition of neutrophil proliferation was identified as a potential strategy to alleviate MVO^36^. A retrospective study by Wang et al. demonstrated that neutrophil count played a role in establishing a no-reflow model^37^, consistent with the outcomes of our study. Recently, SII has been widely applied in clinical research. It was also included among our research variables. However, it was not ultimately incorporated into our prediction model. As a comprehensive inflammatory marker, SII mainly reflects the overall inflammatory response. In contrast, the role of neutrophils in the formation of MVO is more specific and direct. In previous studies, SII has been closely associated with myocardial remodeling after PCI in myocardial infarction patients^38^, an increased risk of in-hospital heart failure^39^, and non-obstructive coronary ischemia^40^.

In addition, FIB is an important response factor of inflammation and thrombosis. Fib is a special acute phase response protein, which has been shown to promote intravascular lipid deposition, regulate the migration and adhesion of inflammatory cells in the blood vessel wall, and promote thrombosis. It is a commonly used coagulation index in clinical practice, and also an important index to predict microangiopathy^41^. According to a study by Nguyen et al., MVO was associated with higher levels of inflammatory biomarkers such as IL-6, fibrinogen, and neutrophil count, especially neutrophils^42^. LVEF is a widely utilized clinical metric for assessing left ventricular systolic function. Previous investigations have indicated an association between MVO and reduced LVEF^43,44^.

**Fig. 6:**
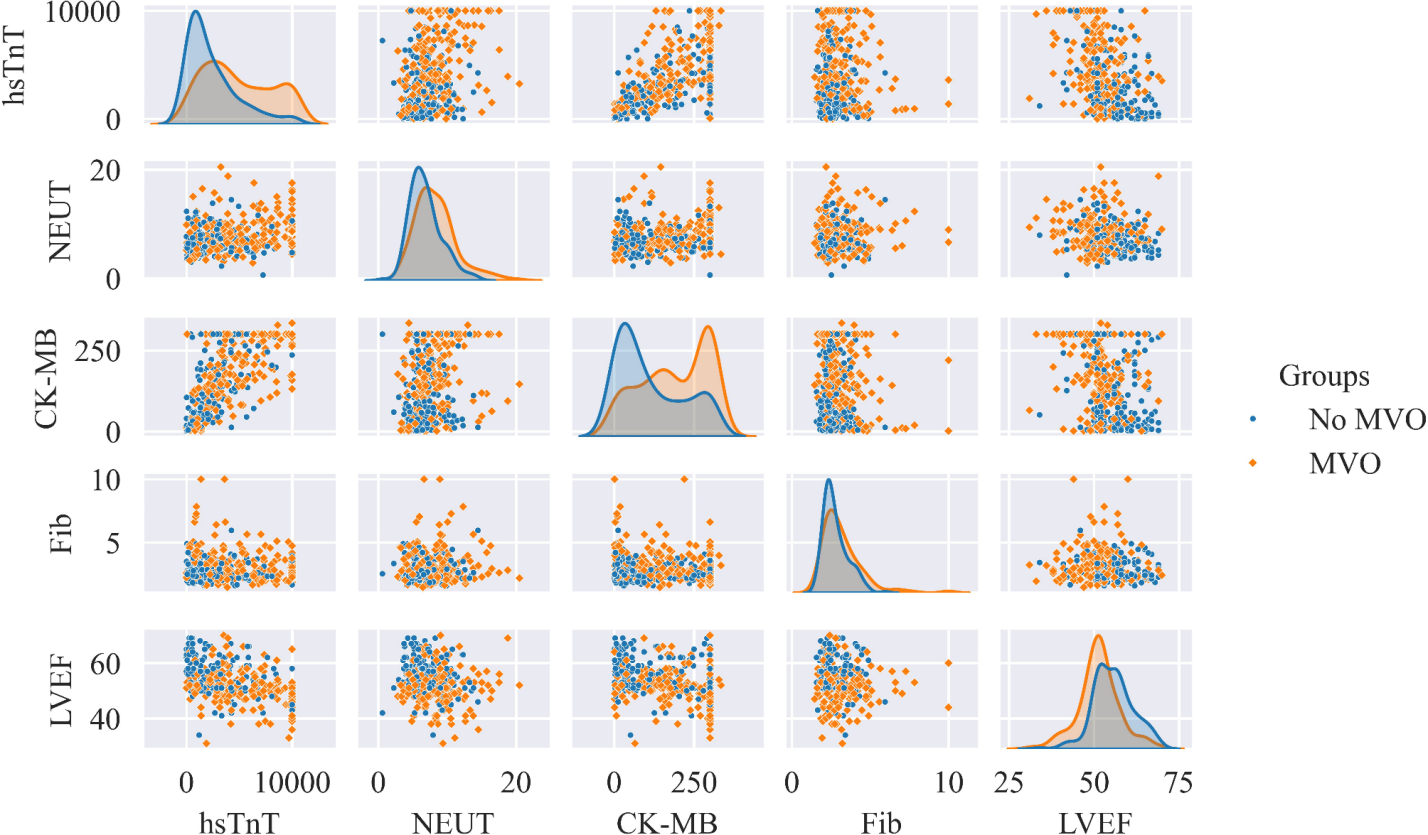
Scatterplot Matrix. Each block on the diagonal is a kernel density estimate plot for the respective variable. The remaining blocks are the scatter plots between each pair of features.

It is noteworthy that the performance of the LR model constructed with the selected features surpasses that of machine learning models, despite the RF-RFE method primarily considering non-linear relationships between variables. This could be attributed to the linear correlation among the features selected. This could be attributed to the linear relationships among the five top features selected and MVO, as illustrated in Fig. 6. The LR model is inherently better suited for uncovering linear patterns among variables. Machine learning models such as Random Forest and XGB are more adept at capturing nonlinear and complex relationships, and their predictive performance might be compromised compared to the simplicity of the LR model.

Compared with previous studies, our study has several innovative aspects. Firstly, it established a multifactor model by synthesizing various patient characteristics, instead of focusing on a single indicator. This more comprehensively reflects the complex factors affecting MVO. Secondly, the automated feature selection using RF-RFE identified the optimal combination of predictive variables, reducing subjective bias and improving model generalization. Thirdly, it systematically compared multiple models and demonstrated the superiority of LR over machine learning models like RF and XGB.

### Limitations

Previous research has highlighted the heightened risk of overfitting when modeling with small sample sizes^45–48^. As a single-center cohort study with a relatively small sample size, it is susceptible to potential biases. Thomas et al. suggested that restricting the hypothesis space of plausible models can help overcome overfitting^45^. In this study, the tree depth for RF, XGB, and GDBT was limited to below 5, and the number of trees was restricted to fewer than 200. The LR model used L2 regularization. Vabalas et al. found that Nested CV and train/test split provide more reliable performance estimates than K-fold Cross-Validation with small sample sizes^46^. To assess the robustness of the model in this study, its performance was evaluated using a prospective cohort dataset entirely independent of the training data.

## Conclusion

Establishing an individualized prediction model for post-PCI MVO using the LR model is feasible. Subsequent work could focus on validating and optimizing the model through an expansion of the sample size and the collection of multicenter data. Additionally, integrating the model into clinical practice and conducting prospective research to optimize perioperative PCI treatment plans will comprehensively validate the clinical application value of this predictive model. We anticipate that cardiovascular digital medicine will play a greater role in refining risk prediction and management for AMI.

## Data Availability

The data, models, and procedures utilized in the article are available to scientific researchers upon reasonable request through the corresponding author. Clinicians can access the model mentioned in the article at https://mvo.model.smilemed.cn.
